# Supplementary material for: Prevalence and Risk Factor Analysis of Post-Intensive Care Syndrome in Patients with COVID-19 Requiring Mechanical Ventilation: A Multicenter Prospective Observational Study
Source: J Clin Med. 2022 Sep 28;11(19):5758. doi: 10.3390/jcm11195758 (PMC9571505; doi:10.3390/jcm11195758)
Supplement: Supplementary file 1 [file jcm-11-05758-s001.zip › jcm-1887899-supplementary.pdf]

Supplemental Table S1. Details of the central office and participating facilities

Central office

TXP Medical Co. Ltd.

#252 Minamikenkyuto, 7-3-1, Hongo, Bunkyo-ku, Tokyo 113-8485, Japan

Participating Facilities

- Asahi General Hospital
- Chiba University Hospital
- Fujita Health University Hospital
- Fukushima Medical University Hospital
- Gifu University Hospital
- Hiroshima University Hospital
- Hitachi General Hospital
- Hyogo Prefectural Kakogawa Medical Center
- Japanese Red Cross Kyoto Daiichi Hospital
- Japanese Red Cross Maebashi Hospital
- Kansai Medical University Medical Center
- Kobe City Medical Center General Hospital
- Kyushu University Hospital
- Nagano Red Cross hospital
- National Hospital Organization Kumamoto Medical Center
- National Hospital Organization Kyoto Medical Center
- National Hospital Organization Tokyo Medical Center
- Osaka General Medical Center
- Osaka Medical and Pharmaceutical University
- Osaka University Hospital
- Saitama Red Cross Hospital
- Shinshu University Hospital
- Tohoku University Hospital
- Tokyo Medical and Dental University Medical Hospital
- Tokyo Metropolitan Geriatric Medical Center
- Tokyo Metropolitan Tama Medical Center
- University Hospital Kyoto Prefectural University of Medicine
- University of the Ryukyus Hospital
- Yao Tokushukai General Hospital
- Yodogawa Christian Hospital
- Yokohama City Minato Red Cross Hospital
- Yokohama City University Medical Center

Supplemental Table S2. All data extraction items

Facility Information

- Hospital type
- Total number of beds
- Number of intensive care unit (ICU) beds and number of ICU beds dedicated to patients with severe coronavirus disease (COVID-19).
- Staff that have direct contact with patients with COVID-19, e.g., physicians, nurses, pharmacists, physiotherapists, dietitians, etc.
- Written ICU protocols, e.g., sedation, administration of analgesics, spontaneous awakening trial, spontaneous breathing trial, assessment of delirium, early rehabilitation, sleep care, and nutrition.
- Assessment tools for delirium, e.g., Confusion Assessment Method for the ICU, Intensive Care Delirium Screening Checklist, etc.
- Visiting hours and online visitation via cell phone, etc.

Variables and measurements

- Basic characteristics (age, sex, body mass index at ICU admission, length of ICU and hospital stay, duration of mechanical ventilation, clinical frailty scale score [enter the status before COVID-19; estimated values are acceptable], ICU mobility scale score on ICU days 3, 5, 7 [enter 0 if the patient is deceased; leave blank if the patient has already been discharged alive], and comorbidities)

Details of comorbidities

Hypertension (use of antihypertensive medication)

Diabetes (HbA1c NGSP≥6·5% and introduction of hypoglycemic agents or insulin)

Cardiac disease (history of angina pectoris, myocardial infarction, valvular heart disease, congestive heart failure, use of medications for heart failure [diuretics, angiotensin-converting enzyme inhibitors, etc.])

Chronic kidney disease (dialysis patient)

Autoimmune disease (history of any autoimmune disease)

Malignant tumors (not only solid tumors, but blood disorders as well)

Chronic obstructive pulmonary disease

Immunodeficiency (immunosuppression, chemotherapy, radiotherapy, administration of steroids [prednisone equivalent: 0·375 mg/kg/day or more]).

- Severity score (Sequential Organ Failure Assessment score at the start of ventilation, A-DROP score on ICU admission; only the total value is acceptable in both cases).
- Laboratory test results (F_IO_2, pH, PaO_2, and $PaCO_2$ results of arterial blood gas analysis before mechanical ventilation and extracorporeal membrane oxygenation [ECMO]. If there is no arterial blood gas analysis result before the start of mechanical ventilation, enter the result obtained at the earliest stage after the start of mechanical ventilation).
- Treatment

To treat or not to treat: reintubation, continuous administration of neuromuscular blocking drugs, prone positioning, ECMO, corticosteroid therapy, renal replacement therapy, duration of rehabilitation program in the ICU by physical therapists, ICU diary (a patient's journal written by staff and family members for several purposes, a record of daily events).

Treatment details: types of tracheostomy, duration from ICU admission to tracheostomy, duration from ICU admission to continuous administration of neuromuscular blocking drug, duration from ICU admission to prone positioning, duration of prone positioning (days), maximum duration per day of prone positioning, duration of ECMO, maximum daily dose of prednisolone equivalent (0 mg/day if no corticosteroids were administered), duration from ICU admission to the start of rehabilitation program.

- Nutritional therapy (in cases where oral intake has already begun, enter the estimated amount. For parenteral nutrition, calories below 5% glucose solution and propofol calories should not be included in the calculation. If not administered during ICU admission, enter 0; if administered after ICU discharge, leave blank) and calories and protein administered for enteral and parenteral nutrition, respectively, from ICU Day 1 to Day 7.
- Delirium and number of ICU days per week under delirium (criteria and diagnostic tools for delirium (Confusion Assessment Method for the ICU; CAM-ICU, Intensive Care Delirium Screening Checklist; ICDSC and others) follow the guidelines of each institution).
- Time period: from ICU death discharge to October 2021, the first survey was for the period from ICU survival discharge to February 2021, and the second survey was for the period from ICU survival discharge to October 2021
- Living with Family.
- Route of infection (familial transmission, other clusters, unknown route).
- Types of personal protective equipment.
- ICU bed configuration (private room, open floor).
- Number of patients received per nurse.

Outcomes

Questionnaire

- Do you feel shortness of breath when you move?
- Do you have difficulty walking?
- Have you lost weight?

- On a scale of 1 to 10 point on the Visual Analog Scale (VAS), how would you rate your physical condition before you were infected with SARS-CoV-2? Scores range between 10 (the patient has reverted to the same state as before the infection) and 0 (the patient is in poor condition after the SARS-CoV-2 infection).
- Do you have trouble remembering things?
- Have you lost the ability to do things you used to be able to do?
- On a scale of 1 to 10 point on the Visual Analog Scale (VAS), how would you rate your cognitive function before you were infected with SARS-CoV-2? Scores range between 10 (the patient has reverted to the same state as before the infection) and 0 (the patient is in poor condition after the SARS-CoV-2 infection).
- Do you feel depressed?
- Do you have anxiety?
- Do you have trouble sleeping?
- On a scale of 1 to 10 point on the Visual Analog Scale (VAS), how would you rate the state of your mental health before you were infected with SARS-CoV-2? Scores range between 10 (the patient has reverted to the same state as before the infection) and 0 (the patient is in poor condition after the SARS-CoV-2 infection).

Barthel index

Short-Memory Questionnaire

Hospital Anxiety and Depression Scale-anxiety

Hospital Anxiety and Depression Scale-depression

EQ-5D-5L

Supplemental Table S3. Number of missing values from patient survey results

Questions	Number of missing values	
	First PICS survey (n=251)	Second PICS survey (n=209)
Dyspnea, n(%)	0 (0)	3 (1.4)
Walking difficulty, n(%)	2 (0.8)	4 (1.9)
Weight loss, n(%)	0 (0)	3 (1.4)
Memory impairment, n(%)	2 (0.8)	1 (0.5)
Execution disability, n(%)	0 (0)	4 (1.9)
Depression, n(%)	0 (0)	2 (1)
Anxiety, n(%)	0 (0)	1 (0.5)
Sleeping disorder, n(%)	0 (0)	1 (0.5)
Visual analogue scale, n(%)		
Physical condition (on a scale of 1 to 10)	1 (0.4)	1 (0.5)
Cognitive function (on a scale of 1 to 10)	2 (0.8)	1 (0.5)
Mental health (on a scale of 1 to 10)	1 (0.4)	1 (0.5)
Barthel Index, n(%)	4 (1.6)	3 (1.4)
Short-Memory Questionnaire, n(%)	5 (2)	3 (1.4)

HADS score, n(%)		
HADS-Anxiety score	1 (0.4)	0 (0)
HADS-Depression score	1 (0.4)	0 (0)
EQ-5D-5L, n(%)	4 (2)	6 (2.9)

HADS; Hospital Anxiety and Depression Scale, PICS; post-intensive care syndrome

Supplemental Table S4. Patient background with both responses to the first and second questionnaires

	n=209
Age, yr, median(IQR)	67(58, 74)
Male, n(%)	167(79.9)
BMI, kg/m^2, median(IQR)	25.0(22.4, 28.3)
SOFA score on the day of ventilation start, median(IQR)	5(3.5, 7)
Clinical frailty scale before hospitalization, median(IQR)	2(1, 2)
Delirium, n(%)	38(18.2)
Duration of delirium within 1 week of ICU admission, day, median(IQR)	2(1, 4)
Duration of invasive mechanical ventilation, day, median(IQR)	9(6, 16)
Length of ICU stay, day, median(IQR)	11(8, 19)
Length of hospital stay, day, median(IQR)	22(13, 40)
Comorbidity, n(%)	
Hypertension	96(45.9)
Diabetes	61(29.2)
Cardiac disease	18(8.6)
Chronic kidney disease	6(2.9)
Autoimmune diseases	7(3.3)
Malignant tumors	10(4.8)
COPD	17(8.1)
Immunodeficiency	5(2.4)
Treatment received during hospital stay	
Reintubation, n(%)	9(4.3)
ECMO, n(%)	25(12)
Duration of ECMO, day, median(IQR)	11(9, 18)
Tracheostomy, n(%)	39(18.7)
Corticosteroid, n(%)	164(78.5)
Maximum prednisolone dose, mg/day, median(IQR)	44(30, 100)
Continuous neuromuscular blocking agent, n(%)	93(44.5)
Prone position, n(%)	113(54.1)
Continuous renal replacement therapy, n(%)	18(8.6)
Rehabilitation program, n(%)	114(54.5)
Time from ICU admission to rehabilitation program initiation, day, median(IQR)	5(2, 16)

BMI body mass index, COPD chronic obstructive pulmonary disease, ECMO extracorporeal membrane oxygenation, ICU Intensive Care Unit, IQR interquartile range, PICS post-intensive care syndrome, SOFA Sequential Organ Failure Assessment

Supplemental Table S5. PICS outcomes for 209 patients who responded to both of the first and second questionnaires

	First PICS survey Assessment of PICS	Second PICS survey Assessment of PICS	p value
PICS, n(%)	123(58.9)	127(60.8)	0.67
Physical impairment	40(19.1)	39(18.7)	1.0
Cognitive impairment	100(47.8)	111(53.1)	0.13
Mental disorder	66(31.6)	60(28.7)	0.32
Dyspnea, n (%)	104(49.8)	96(45.9)	0.47
Walking difficulty, n (%)	75(35.9)	54(25.8)	0.001
Weight loss, n (%)	127(60.8)	48(23)	<0.001
Memory impairment, n (%)	61(29.2)	66(31.6)	0.52
Executive dysfunction, n (%)	100(47.8)	93(44.5)	0.77
Depression, n (%)	89(42.6)	81(38.8)	0.5
Anxiety, n (%)	123(58.9)	107(51.2)	0.04
Sleeping disorder, n (%)	92(44)	92(44)	1.0
Visual analog scale, median(IQR)			
Physical condition (on a scale of 1 to 10)	7.3(5.5, 8.6)	7.4(6.2, 8.7)	0.36
Cognitive function (on a scale of 1 to 10)	8.9(7.4, 9.9)	8.6(7.1, 9.9)	0.06
Mental health (on a scale of 1 to 10)	8.3(6.1, 9.5)	8(6.5, 9.4)	0.33
Barthel Index, median(IQR)	100(99, 100)	100(95, 100)	0.46
Short-Memory Questionnaire, median(IQR)	40(34, 44)	39(34, 43)	0.002
HADS score, median(IQR)	8(3, 14)	7(3, 14)	0.24
HADS-Anxiety score	4(1, 7)	3(1, 7)	0.09
HADS-Depression score	4(1, 7)	4(1, 7)	0.57
EQ-5D-5L, median(IQR)	0.828(0.708, 1)	0.844(0.759, 1)	0.04

HADS; Hospital Anxiety and Depression Scale, IQR; interquartile range, PICS; post-intensive care syndrome

Supplemental Table S6. Patient background of 42 dropout patients before the second PICS survey

	n=42
Age, yr, median(IQR)	66.5(57, 75)
Male, n(%)	33(78.6)
BMI, kg/m^2, median(IQR)	26.9(22.6, 29.7)
SOFA score on the day of ventilation start, median(IQR)	5.5(4, 7)
Clinical frailty scale before hospitalization, median(IQR)	2(1, 3)
Delirium, n(%)	12(28.6)
Duration of delirium within 1 week of ICU admission, day, median(IQR)	2(1, 4)
Duration of invasive mechanical ventilation, day, median(IQR)	9(6, 16)
Length of ICU stay, day, median(IQR)	11(8, 22)
Length of hospital stay, day, median(IQR)	29(13, 43)
Comorbidity, n(%)	
Hypertension	18(42.9)
Diabetes	19(45.2)
Cardiac disease	8(19)
Chronic kidney disease	1(2.4)
Autoimmune diseases	2(4.8)
Malignant tumors	4(9.5)
COPD	4(9.5)
Immunodeficiency	3(7.1)
Treatment received during hospital stay	
Reintubation, n(%)	1(2.4)
ECMO, n(%)	8(19)
Duration of ECMO, day, median(IQR)	15(8, 17)
Tracheostomy, n(%)	12(28.6)
Corticosteroid, n(%)	30(71.4)
Maximum prednisolone dose, mg/day, median(IQR)	44(0, 75)
Continuous neuromuscular blocking agent, n(%)	19(45.2)
Prone position, n(%)	19(45.2)
Continuous renal replacement therapy, n(%)	1(2.4)
Rehabilitation program, n(%)	26(61.9)
Time from ICU admission to rehabilitation program initiation, day, median(IQR)	4(2, 11)

BMI body mass index, COPD chronic obstructive pulmonary disease, ECMO extracorporeal membrane oxygenation, ICU Intensive Care Unit, IQR interquartile range, PICS post-intensive care syndrome, SOFA Sequential Organ Failure Assessment
